# A Single Session of Bifrontal tDCS Can Improve Facial Emotion Recognition in Major Depressive Disorder: An Exploratory Pilot Study

**DOI:** 10.3390/biomedicines10102397

**Published:** 2022-09-26

**Authors:** Laetitia Imbert, Rémi Moirand, Benoit Bediou, Olivier Koenig, Gabrielle Chesnoy, Eric Fakra, Jérôme Brunelin

**Affiliations:** 1 PSYR2 Team, Lyon Neuroscience Research Center, INSERM, U1028, CNRS, UMR5292, F-69000 Lyon, France or; 2 Université Claude Bernard Lyon 1, Lyon University, F-69100 Villeurbanne, France; 3 Pôle EST, Centre Hospitalier Le Vinatier, F-69500 Bron, France; 4 Faculté de Psychologie et Sciences de l’Éducation, Campus Biotech, Université de Genève, 9 chemin des mines, CH-1211 Geneva, Switzerland; 5 Laboratoire d’Étude des Mécanismes Cognitifs, Équipe d’Accueil 3082, Université Lyon 2, Université de Lyon, F-69500 Bron, France; 6 Université Jean Monnet, Centre Hospitalier Universitaire, F-42000 Saint Etienne, France

**Keywords:** facial emotion recognition, tDCS, brain stimulation, depression, DLPFC, MDD

## Abstract

Emotional processing deficits are key features in major depressive disorder (MDD). Neuroimaging studies indicate that the dorsolateral prefrontal cortex (DLPFC) plays a pivotal role in both depressive symptoms and emotional processing. Recently, transcranial Direct Current Stimulations (tDCS) applied over the DLPFCs have held the promise to alleviate the symptoms in patients with MDD, but the effect on emotional processing in the patients is unclear. Here, we investigated the effect of a single session of tDCS over the DLPFCs on the emotional processing in patients with treatment-resistant MDD. In a randomized sham-controlled study, 35 patients received a single 30 min session of either active (2 mA, *n* = 18) or sham tDCS (*n* = 17). The anode was placed over the left and the cathode over the right DLPFC. Emotional processing accuracy was measured by a facial emotion recognition (FER) task. We observed an overall improvement in FER performance after the active tDCS, but not the sham tDCS. These exploratory results suggest that a single session of tDCS over the DLPFCs may improve FER in MDD, a crucial function of social cognition. Further studies are needed to investigate whether this acute improvement of FER in response to a single tDCS session could translate into clinical benefits or predict remission following repeated sessions of stimulation.

## 1. Introduction

Major depressive disorder (MDD) is the most common psychiatric disease. In 2017, over 300 million people were estimated to suffer from depression, which corresponds to 4.4% of the world’s population [1]. Clinical symptoms and cognitive deficits associated with MDD largely affect the social functioning of patients and contribute to the global burden associated with the disease. Patients with MDD often display pessimistic thoughts, increased self-attention, exhibit excessive processing and over-interpret negative emotional stimuli [2]. All these deficits promote negative self-related information and prolonged negative emotions leading to the onset and the maintenance of depressive episodes [3,4]. Among the abnormalities in the emotional processing in patients with MDD, facial emotion recognition (FER), a key feature of social interaction, has been repeatedly observed to be impaired, as evidenced by a lower global performance for both positive and negative emotions [5,6,7].

Despite many years of research, the brain network that underlies FER deficits in MDD remains unclear and may result from abnormal connectivity within widespread brain networks. Neuroimaging studies have suggested imbalanced activity within several cortical and subcortical brain regions, with a hypoactive dorsolateral prefrontal cortex (DLPFC) rendering altered top-down inhibitory control, thereby leading to hyperactivity within the ventral anterior cingulate cortex, the insula, and the amygdala [2,8]. Otherwise, in patients with MDD, a hypoactivity in the left DLPFC with regards to hyperactivity in the right DLPFC has been linked to the severity of symptoms [9].

This imbalanced activity within the right and left DLPFC [10] has been considered the hallmark of the hypothetical basis for the use of noninvasive brain stimulation (NIBS) as an alternative therapy for treatment-resistant symptoms in MDD [11]. In this line, some authors have proposed to target either the left DLPFC with high frequency repetitive transcranial magnetic stimulation [12] or the right DLPFC with low frequency stimulation [13]. The authors have proposed to use a bifrontal transcranial direct current stimulation (tDCS) with the anode over the left DLPFC coupled to the cathode over the right DLPFC, to examine whether restoring this dysbalanced activity would in turn decrease depressive symptoms. They have reported a beneficial effect of repeated sessions of tDCS with this electrode set-up on depressive symptoms [14]. Although the real advantageous effects of tDCS in clinical settings remain unclear, beneficial clinical effects of bifrontal tDCS in MDD have been corroborated by recent large randomized clinical trials, and are supported by meta-analyses and international evidence-based guidelines [15,16,17]. However, while numerous studies have supported the clinical effect of tDCS to decrease symptoms and increase cognitive functioning in MDD [18], only a few studies have investigated whether tDCS can alleviate FER deficits, whereas the same brain regions appear to be involved in both processes [11,19]. To our knowledge, although some studies have been conducted with healthy volunteers, no sham-controlled, 2-arm parallel tDCS study has directly investigated the effect of bifrontal tDCS on FER in patients with MDD. Only one cross-over study has supported a beneficial effect of a single tDCS session at 1.5 mA on FER, with the anode over the left DLPFC and the cathode over the right supraorbital region. However, this study used an online design where the stimulation was delivered during the FER task, which did not allow for an examination of whether the observed beneficial effect outlasted the stimulation period [20].

Here, we propose to Investigate the acute effects of a single (30 min, 2 mA) session of bifrontal tDCS, with the anode over the left DLPFC and the cathode over the right DLPFC, on FER performance in patients with MDD. We hypothesize that an active tDCS would result in an increase in FER abilities compared with a sham stimulation.

## 2. Materials and Methods

### 2.1. Sample

In a double-blind, sham-controlled study, 40 patients with a non-psychotic unipolar major depressive episode, according to the DSM-V criteria, were recruited in our specialized unit for treatment-resistant depression at CH le Vinatier psychiatric hospital. The diagnosis was determined by a trained psychiatrist during a structured clinical interview with the Mini International Neuropsychiatric Interview M.I.N.I. 7.0. In the current study, depression was considered as treatment-resistant when at least two trials with antidepressants from different pharmacological classes with adequate doses and durations failed to produce a significant clinical improvement. Concomitant actual or past other psychiatric diagnoses were exclusion criteria (except for tobacco use disorder and past anxious disorders).

Patients had to present disabling depressive symptoms, despite stable antidepressant medication for at least 4 weeks at an adequate dosage. They had to present a mild to severe depression evaluated by a 10-item Montgomery–Åsberg Depression Rating Scale (MADRS) score greater than or equal to 20. All the participants signed a written informed consent after a description of the study. The study was approved by an ethics committee on 13 April 2016 (CPP sud Est III, Lyon, France, registration number 2016-019B; French national health authorities ANSM 2016-A00415-46). The study was pre-registered in a public database (www.clinicaltrials.gov) before its completion on 8 June 2016 (registration number NCT02793258).

Patients were randomly assigned to receive either an active (*n* = 20) or a sham tDCS (*n* = 20). The allocation was provided by the sponsor of the study (CH le Vinatier, randomization in a 1:1 ratio by blocks with a block size of 4, no stratification). Due to technical issues during the recording of the results or the completion of the task, five patients were not included in the analysis: two patients from the active group (no post-tDCS evaluation) and 3 from the sham group (2 pre-tDCS, 1 post-tDCS missing data). Thus, the final analyzed sample consisted of 18 patients in the active group and 17 patients in the sham group.

### 2.2. Stimulation Procedure

Stimulation sessions were delivered by using a commercial NeuroConn DC Stimulator Plus (Neurocare, Ilmeneau, Germany) with two 7 × 5 cm (35 cm^2^) electrodes in saline-soaked sponges (0.9% NaCl). The anode was placed over the left DLPFC (F3 according to the international EEG 10-20 system electrode placement system) and the cathode over the right DLPFC (F4).

For the active tDCS session, the intensity was set at 2 mA and lasted for 30 min (ramp up/down, 30 s). The sham stimulation was delivered using the commercial “STUDY mode” of the NeuroConn device and consisted of delivering 60 s of active stimulation (ramp up/down, 30 s) followed by no stimulation during the remaining 30 min of the stimulation period. There were only brief pulses of 110 μA every 550 ms to control the impedance and keep the manipulator blind to the condition. During the stimulation sessions, the patients were comfortably seated in a room dedicated to brain stimulation. To control for the influence of brain state during the stimulation procedure [21], patients were required to feel the most neutral emotion as possible during the tDCS sessions. Moreover, in order to avoid any influence of the variation of circadian rhythms, the stimulation sessions were given at the same time, most often on Monday morning at 10:00 a.m. The treatment was administered by trained nurses who were blinded to the treatment allocations. The blinding of the nurses was ensured with a unique study code number allocated to each participant and with the device that displayed a continuous measure of impedance during the stimulation sessions.

### 2.3. Facial Emotion Recognition Task

The FER task required participants to categorize faces according to their expressions into one of the six basic emotions (happiness, sadness, fear, disgust, anger and surprise). To be more ecological, and based on previous works showing that it is more sensitive and discriminative to identify cognitive biases based on moderate intensities, intensities of 20%, 40%, 50%, 60% and 80% were presented [22,23]. The stimuli were obtained by morphing between neutral (0% expression) and expressive (100%) faces from Ekman’s validated database. The task consisted of presenting 8 identities per emotion (4 males and 4 females) at each intensity, for a total of 240 stimuli presented in a randomized order. Prior to the task, all participants underwent a 36-trial practice block in which emotions were presented at 100% intensity in order to familiarize participants with the stimuli used.

**Figure 1:**
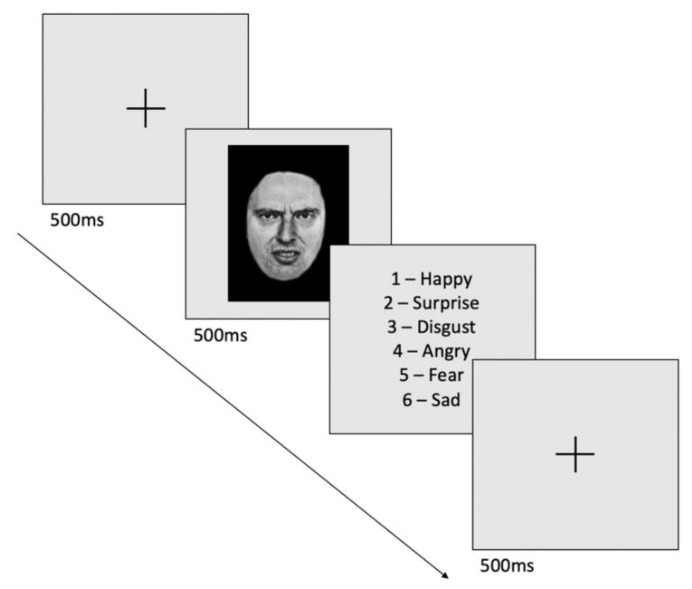
Illustration of the facial emotion recognition task. Faces expressing emotions at different intensities (happy, surprise, disgust, angry, fear or sad) were successively presented for 500 ms on a computer screen preceded by a fixation cross (500 ms).

The task was presented as follows: a central fixation cross was displayed for 500 ms, followed by the stimulus for 500 ms (the picture of the face was placed in the middle and covered about 1/4 of the screen). Participants were then required to identify the emotion as quickly and as accurately as possible, by pressing the key on a keyboard corresponding to one of the 6 emotions. Finally, a blank screen was presented for 550 ms between the response and the start of a new trial. Twenty minutes were required to complete the task (Figure 1). The participant was placed at about 60 cm from the screen. The participants were asked to perform the task twice in the same day, one before the stimulation session (usually between 9.00 and 10.00 a.m.) and a second time immediately after the end of the tDCS session (30 min to one hour later).

### 2.4. Statistical Analysis

All statistical analyses were carried out using JASP software (created by Eric-Jan Wagenmakers, version 0.12.2, Amsterdam, The Netherlands) by an experimenter blinded to the treatment allocations. The alpha threshold was set at 0.05. For all statistical tests used in this study, parametric tests were used only if the conditions of application were met, otherwise non-parametric versions were used. Baseline clinical and socio-demographic characteristics were compared using Student’s t- or Mann–Whitney U tests and Fisher’s exact tests.

The primary outcome of the study was the change in accuracy to the FER task (the proportion of correct responses for each emotion, averaged across morphing intensities). A 3-way mixed standard repeated-measures ANOVA was undertaken to compare the effect of the groups (the sham and active tDCS) as between-subject factors, and emotions (anger, disgust, fear, happy, sad and surprise) and sessions (pre- and post-tDCS) as within-subject factors. Further analyses were performed to investigate the change in overall emotion recognition accuracy using two separate 2-factor repeated-measures ANOVAs (one by group): session and emotion. Exploratory analyses were carried out using post hoc *t*-tests with a Holm’s correction to investigate the change in the FER accuracy for each emotion, emotion by emotion. In this study, morphing intensities, identities and sex were not introduced as covariates in the analyses, but were used as tools to increase the sensitivity and the ecological validity of the task.

## 3. Results

### 3.1. Clinical and Sociodemographic Characteristics

No difference was observed between patients from the active and the sham groups at inclusion regarding sex, age, education level, duration of the current episode, illness duration and MADRS scores at baseline (see Table 1).

The tDCS session was well tolerated, and no serious adverse events were observed throughout the study period. Only itching sensations were described by some patients in both groups.

### 3.2. Effects of tDCS on Facial Emotion Recognition

#### 3.2.1. Within-Group Analysis

The repeated-measures ANOVA on recognition accuracy (measured as the frequency of correct responses) revealed a trend toward significance for the emotion*session*group interaction (F_(5,165)_ = 2.231; η^2^ = 0.062; *p* = 0.05).

#### 3.2.2. Between-Group Analysis

**Figure 2:**
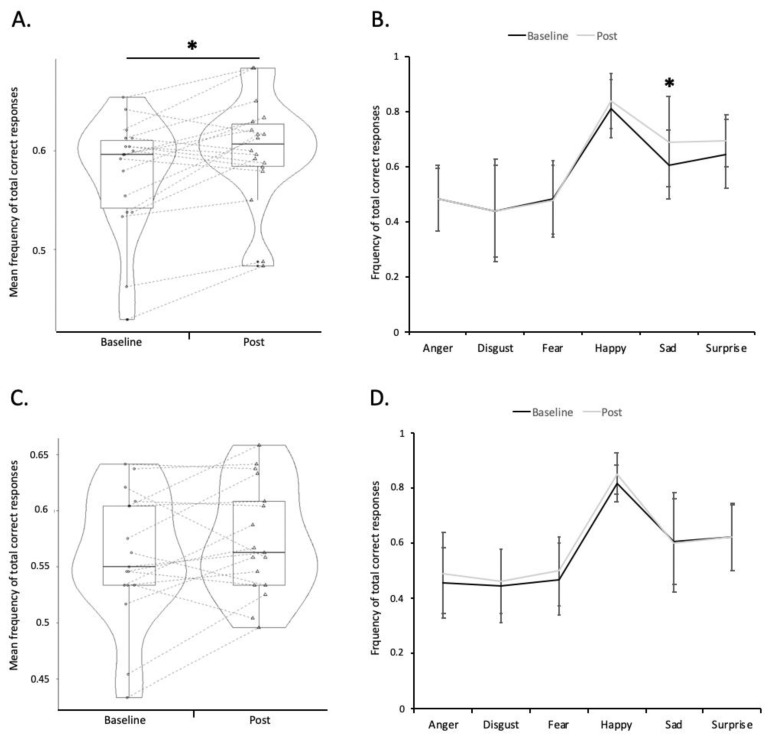
Changes in mean correct recognition of facial emotion expression following active (panel A) or sham tDCS (panel B). The repeated-measures ANOVA on recognition accuracy (measured as the frequency of correct responses) revealed a trend toward significance for the emotion*session*group interaction (F_(5,165)_ = 2.231; η^2^ = 0.062; *p* = 0.05). (**A**) significant overall facial emotion recognition (FER) improvement following active tDCS (ANOVA emotion*session, *p* = 0.04); (**B**) significant improvement of sad faces recognition after active tDCS (post hoc t-test with Holm’s *: *p*-value < 0.05); mean frequency of total correct responses emotion by emotion (±standard deviation) at baseline (black) and post-tDCS (grey). (**C**) no significant effect of sham tDCS on overall FER performance: (*p* = 0.08). (**D**) no significant effect of sham tDCS emotion by emotion.

To follow up on the three-way interaction, two separate two-factor repeated-measures ANOVAs (session*emotion) were carried out, one in each group (see Figure 2).

These ANOVAs revealed a significant effect of emotion in the active group (F_(5,85)_ = 25.689; η^2^ = 0.602; *p* < 0.01), and in the sham group (F_(5,80)_ = 28.495; η^2^ = 0.640; *p* < 0.01).

Regarding the effect of the sessions (the effect of the tDCS), a significant +2.6% overall improvement in FER performance between the baseline and after the active tDCS was observed (95% confidence interval (95% CI) = [−0.042; −0.009]; F_(1,17)_ = 11.026; η^2^ = 0.393; *p* < 0.01) (Figure 2A). Conversely, no significant effect of the session was observed in the sham group: +2.0% (95% CI = [−0.042; 0.002]; F_(1,16)_ = 3.587; η^2^ = 0.183; *p* = 0.08;) (Figure 2C).

Finally, the effect of the session*emotion interaction was significant in the active group (F_(5,85)_ = 2.399; η^2^ = 0.124; *p* = 0.04), but not in the sham group (F_(5,80)_ = 0.497; η^2^ = 0.030; *p* = 0.78).

#### 3.2.3. Exploratory Analysis

To further examine how active tDCS affects FER differentially depending on the emotion, we then compared the performance between pre- and post-tDCS by emotion (averaging across intensity levels to increase sensitivity) using the *t*-test with Holm’s correction. The analyses revealed a significant +8.2% (95% CI = [−0.015; −0.007]; t = −3.812; *p* < 0.01) improvement in the frequency of correct responses for “sad” face recognition in the active tDCS group (Figure 2B). No other significant results were found on the five other emotions in the active group. No significant effect of the sham tDCS was observed on any of the six emotions (Figure 2D).

Importantly, the improvement in sadness recognition did not occur at the expense of an increased misinterpretation of other faces as sad. Indeed, we observed no significant difference in the number of false alarms (number of faces misinterpreted as sad) among the sessions of active tDCS (t = −0.856; df = 17; Cohen’s d = −0.202; 95% CI [−0.666; 0.268]; *p* = 0.40), suggesting an overall increase in sad recognition without any increase in sad sensitivity, excluding a negative impact of tDCS that could have reflected an increased overinterpretation of all faces being misinterpreted as sad.

## 4. Discussion

In this randomized sham-controlled pilot study, we found a significant overall improvement in FER performance in patients with MDD after a single session of active tDCS, for both positive and negative emotions. Given the exploratory nature of this pilot study, no *a priori* sample size calculation was performed, and the power of the current study is limited. The size of the sample is, however, comparable with other studies that have investigated the effects of noninvasive brain stimulation on FER in patients with MDD (17 patients in Brennan et al., 2017 [20]; 24 in de Moraes et al., 2020 [24]). Although promising, these preliminary results should thus be taken with caution and require further replication before drawing any strong conclusions. Only a marginal improvement in FER performance after the tDCS sham session was reported, suggesting that the improvement in FER performance in the active group could be causally attributed to the effect of the stimulation, not to a learning effect between sessions. The marginal improvement in the control group may reflect a test-retest effect and confirms the importance of including a control group in order to draw causal conclusions based on the expected statistical interaction. Exploratory post hoc investigations suggested that this effect was mainly driven by an increased performance in the recognition of sad faces after the active tDCS. Importantly, the improvement in sadness recognition did not appear to be related to a negative impact of tDCS that might have resulted in an increased sensitivity to sad faces (i.e., an excessive misinterpretation of all emotional faces as sad), as we did not observe a significant difference in the number of false alarms (the number of faces misinterpreted as sad) among the tDCS sessions. This improvement in sad faces recognition was in line with previous studies demonstrating better sad faces recognition performances in patients who had achieved remission as compared with patients with acute depressive episodes [25].

These results are in line with other sham-controlled studies that have reported a beneficial effect of a single session of NIBS over the left DLPFC on FER in patients with different psychiatric disorders. In a first study conducted on 17 patients with MDD, Brennan and colleagues [20] reported that an online tDCS (1.5 mA delivered during the FER task) could improve the recognition of both positive (happy) and negative (anger) emotions. In this study, the anode was placed over the left DLPFC and the cathode over the contralateral supraorbital area, which differs from the bifrontal electrode set-up used in our study. Using an offline design, a beneficial effect of tDCS on overall FER performance was observed in patients with schizophrenia with the anodal tDCS over the left DLPFC coupled with the cathodal over the right upper arm [26]. Finally, a single session of high frequency rTMS over the left DLPFC induced an increased sensitivity for happy faces in patients with MDD [24]. Combined with ours, these results support a key role of the left DLPFC in FER and suggest that stimulating this brain region with NIBS in patients with FER deficits would lead to beneficial outcomes in social cognition. Further studies are needed to determine the optimal stimulation parameters in order to obtain a greater effect.

If one can hypothesize that stimulating the left DLPFC is sufficient to modulate FER in MDD, it is important to note that the effects of tDCS are not limited to the brain regions below the electrodes, but can spread through the interconnected cortical regions modulating resting state networks [27], reaching deeper structures and modifying the subcortical dopamine transmission [28]. Moreover, electric field modelling studies have suggested that the position of the two electrodes matters for the current distribution in the cortex. With the electrode montage we used (anodal F3, cathodal F4), the peak of the induced electric field was situated in the medial PFC [29], corroborating imaging studies that have observed the involvement of this brain region in FER [30]. An active control condition combined with electric field modelling would have allowed us to investigate some of the mechanisms by which tDCS may affect facial emotion recognition. Especially, such an active control condition applied over a control brain region should allow us to decipher whether the targeted region (i.e., the DLPFC) plays a specific critical role, or not, on the observed effects.

The overall improvement on the FER task, a key feature of social cognition [31], observed after a single session of tDCS, would indicate that tDCS might allow the improvement of interaction and communication with others, thus leading to a better understanding of the surrounding environment. This is of major importance since abnormalities in social cognition have been involved in relapses in MDD [32]. Addressing social cognition to improve the interaction and communication with others should be considered in the treatment of MDD, as these aspects are not always improved after conventional treatments [33]. It seems of importance to propose a therapy that could act on these two fundamental aspects to achieve remission and prevent a relapse in MDD. In this line, tDCS may be a useful therapeutic strategy since the repeated sessions of tDCS, using the same electrode set-up as suggested in the current study, hold the promise of being a treatment for depressive symptoms [17,34]. Furthermore, the repeated sessions of tDCS combined with cognitive emotion training may also improve FER [35]. The tDCS could therefore be a very attractive treatment solution alleviating both the deficits in social cognition and the clinical symptoms.

It should be noted that the current results also show a very high inter-individual heterogeneity in the response pattern to the tDCS session (Figure 2A). This heterogeneity has been repeatedly observed in the field of NIBS, for both clinical and cognitive outcomes. Further studies investigating predictive markers of response on the clinical, cognitive and social levels are highly needed to rapidly identify patients who could benefit from a tDCS session [36]. This is of major importance to avoid giving a false hope to treatment-resistant patients with long histories of depression, and to avoid engaging them in time-consuming protocols [37]. In line with the studies developed by Harmer and colleagues, further studies are needed and encouraged to determine whether the early changes in FER after a single NIBS session could predict future clinical improvement following repeated sessions. Indeed, this seems of major interest since it has been reported that restoring emotional processing at the behavioral and neural level may be a precursor to clinical improvements after medication-based antidepressant treatment [38,39].

Some limitations should be acknowledged. The main limitation of the current study is relative to the borderline statistical significance of the primary analysis, leading us to investigate the effect of the tDCS on each arm separately. Secondly, despite the double-blind design of the current study, we did not systematically evaluate the quality of the blinding for neither the participants, the clinical raters nor the tDCS manipulator, while it has been recently well described that the sham procedure is an important factor in the tDCS field [40]. For instance, the tDCS manipulator might observe redness under the electrode only in patients from the active group. However, in order to limit this potential bias in the current study, the person who analyzed the data was not in contact with the patients during the stimulation sessions and had no information about their group assignments.

Finally, one may hypothesize that tDCS could have an effect on the overall basic cognitive functioning in patients leading to FER improvements [18]. We did not test whether such an increase in the basic cognitive functioning may lead to an indirect beneficial effect on the observed improvement in FER in the current study. Investigating social cognition with a laboratory FER task has some drawbacks that prevent us from making a direct translation from what is observed in the lab to what happens in real life. We cannot rule out that the explicit emotion recognition task we used may itself affect the emotion recognition capacities of the participants through a feedback loop effect. The feedback loop effect could modulate the general emotional state of the participants, their intrinsic capacities of recognizing one’s emotional expression, as well as their own emotional states. However, to increase the ecological validity of our task, we used different identities, sex and facial expressions with varying emotional intensities (i.e., from 20 to 80%) and all the stimuli we used were only presented one time to avoid a blunted effect on emotions.

## 5. Conclusions

Although preliminary, the presented results suggest a beneficial effect of a single tDCS session on FER performance as compared to a sham in MDD. These results support the implication of the DLPFC in FER. However, the statistical power and the exploratory nature of our study did not allow us to draw any clear conclusions regarding the usefulness of tDCS on FER in clinical settings. The use of tDCS with the proposed parameters requires further replication.

## Figures and Tables

**Table 1:** Clinical and sociodemographic characteristics of the population included in the analysis.

	Sham tDCS	Active tDCS	*p*
*n* (male/female)	17 (5/12)	18 (9/9)	0.36
Age	51.0 (±9.8)	48.4 (±9.1)	0.42
Education level (years)	15.2 (±3.1)	13.6 (±2.6)	0.11
Current episode duration (months)	16.6 (±14.9)	23.6 (±14.9)	0.14
Illness duration (years)	23.1 (±13.9)	23.7 (±12.2)	0.90
MADRS_10_ (baseline score)	27.2 (±5.0)	26.9 (±4.8)	0.86
Antidepressant medication (*n*)			
SSRIs	11	10	0.73
SNRIs	5	6	1.00
TCAs	6	5	0.73
MAOIs	1	1	1.00
Other medication (*n*)			
benzodiazepine	3	3	1.00
antipsychotics	2	3	1.00

Results are given as mean (±standard deviation), *p*: student *t*-test or Mann–Whitney U test, Fisher’s exact test for gender and medication. MADRS_10_: Montgomery– Asberg Depression Rating Scale; tDCS: transcranial Direct Current Stimulation; medication: SSRIs: selective serotonin reuptake inhibitors; SNRIs: serotonin and norepinephrine reuptake inhibitors; TCAs: tricyclic antidepressants; MAOIs: monoamine oxidase inhibitors.

## Data Availability

The data that support the findings of this study are available on request from the corresponding author (J.B.).

## References

[1] World Health Organization (2017). Depression and Other Common Mental Disorders: Global Health Estimates. https://apps.who.int/iris/bitstream/handle/10665/254610/W?sequence=1.

[2] Disner S.G., Beevers C.G., Haigh E.A.P., Beck A.T. (2011). Neural Mechanisms of the Cognitive Model of Depression. Nat. Rev. Neurosci..

[3] Fossati P. (2018). Is Major Depression a Cognitive Disorder?. Rev. Neurol..

[4] Gotlib I.H., Joormann J. (2010). Cognition and Depression: Current Status and Future Directions. Annu. Rev. Clin. Psychol..

[5] Kohler C.G., Hoffman L.J., Eastman L.B., Healey K., Moberg P.J. (2011). Facial Emotion Perception in Depression and Bipolar Disorder: A Quantitative Review. Psychiatry Res..

[6] Dalili M.N., Penton-Voak I.S., Harmer C.J., Munafò M.R. (2015). Meta-Analysis of Emotion Recognition Deficits in Major Depressive Disorder. Psychol. Med..

[7] Delle-Vigne D., Wang W., Kornreich C., Verbanck P., Campanella S. (2014). Emotional Facial Expression Processing in Depression: Data from Behavioral and Event-Related Potential Studies. Neurophysiol. Clin./Clin. Neurophysiol..

[8] Phillips M.L., Drevets W.C., Rauch S.L., Lane R. (2003). Neurobiology of Emotion Perception I: The Neural Basis of Normal Emotion Perception. Biol. Psychiatry.

[9] Grimm S., Beck J., Schuepbach D., Hell D., Boesiger P., Bermpohl F., Niehaus L., Boeker H., Northoff G. (2008). Imbalance between Left and Right Dorsolateral Prefrontal Cortex in Major Depression Is Linked to Negative Emotional Judgment: An FMRI Study in Severe Major Depressive Disorder. Biol. Psychiatry.

[10] George M.S., Wassermann E.M., Kimbrell T.A., Little J.T., Williams W.E., Danielson A.L., Greenberg B.D., Hallett M., Post R.M. (1997). Mood Improvement Following Daily Left Prefrontal Repetitive Transcranial Magnetic Stimulation in Patients With Depression: A Placebo-Controlled Crossover Trial. AJP.

[11] Gibson B.C., Vakhtin A., Clark V.P., Abbott C.C., Quinn D.K. (2022). Revisiting Hemispheric Asymmetry in Mood Regulation: Implications for RTMS for Major Depressive Disorder. Brain Sci..

[12] O’Reardon J.P., Solvason H.B., Janicak P.G., Sampson S., Isenberg K.E., Nahas Z., McDonald W.M., Avery D., Fitzgerald P.B., Loo C. (2007). Efficacy and Safety of Transcranial Magnetic Stimulation in the Acute Treatment of Major Depression: A Multisite Randomized Controlled Trial. Biol. Psychiatry.

[13] Brunelin J., Jalenques I., Trojak B., Attal J., Szekely D., Gay A., Januel D., Haffen E., Schott-Pethelaz A.-M., Brault C. (2014). The Efficacy and Safety of Low Frequency Repetitive Transcranial Magnetic Stimulation for Treatment-Resistant Depression: The Results From a Large Multicenter French RCT. Brain Stimul..

[14] Brunoni A.R., Valiengo L., Baccaro A., Zanão T.A., de Oliveira J.F., Goulart A., Boggio P.S., Lotufo P.A., Benseñor I.M., Fregni F. (2013). The Sertraline vs Electrical Current Therapy for Treating Depression Clinical Study: Results From a Factorial, Randomized, Controlled Trial. JAMA Psychiatry.

[15] Fregni F., El-Hagrassy M.M., Pacheco-Barrios K., Carvalho S., Leite J., Simis M., Brunelin J., Nakamura-Palacios E.M., Marangolo P., Venkatasubramanian G. (2021). Evidence-Based Guidelines and Secondary Meta-Analysis for the Use of Transcranial Direct Current Stimulation in Neurological and Psychiatric Disorders. Int. J. Neuropsychopharmacol..

[16] Moffa A.H., Martin D., Alonzo A., Bennabi D., Blumberger D.M., Benseñor I.M., Daskalakis Z., Fregni F., Haffen E., Lisanby S.H. (2020). Efficacy and Acceptability of Transcranial Direct Current Stimulation (TDCS) for Major Depressive Disorder: An Individual Patient Data Meta-Analysis. Prog. Neuro-Psychopharmacol. Biol. Psychiatry.

[17] Brunoni A.R., Moffa A.H., Sampaio-Junior B., Borrione L., Moreno M.L., Fernandes R.A., Veronezi B.P., Nogueira B.S., Aparicio L.V.M., Razza L.B. (2017). Trial of Electrical Direct-Current Therapy versus Escitalopram for Depression. N. Engl. J. Med..

[18] Mondino M., Bennabi D., Poulet E., Galvao F., Brunelin J., Haffen E. (2014). Can Transcranial Direct Current Stimulation (TDCS) Alleviate Symptoms and Improve Cognition in Psychiatric Disorders?. World J. Biol. Psychiatry.

[19] Psomiades M., Fonteneau C., Suaud-Chagny M.-F., Haesebaert F., Brunelin J. (2016). Neurostimulation du cortex préfrontal dorsolatéral: Quels effets sur la symptomatologie, l’humeur et les émotions dans la dépression et la schizophrénie?. St. Ment. Québec.

[20] Brennan S., McLoughlin D.M., O’Connell R., Bogue J., O’Connor S., McHugh C., Glennon M. (2017). Anodal Transcranial Direct Current Stimulation of the Left Dorsolateral Prefrontal Cortex Enhances Emotion Recognition in Depressed Patients and Controls. J. Clin. Exp. Neuropsychol..

[21] Silvanto J., Pascual-Leone A. (2008). State-Dependency of Transcranial Magnetic Stimulation. Brain Topogr.

[22] Kohler C.G., Turner T.H., Bilker W.B., Brensinger C.M., Siegel S.J., Kanes S.J., Gur R.E., Gur R.C. (2003). Facial Emotion Recognition in Schizophrenia: Intensity Effects and Error Pattern. AJP.

[23] Surguladze S.A., Young A.W., Senior C., Brébion G., Travis M.J., Phillips M.L. (2004). Recognition Accuracy and Response Bias to Happy and Sad Facial Expressions in Patients With Major Depression. Neuropsychology.

[24] de Moraes R., Pereira da Cruz R., Manso Melchiades A., Arantes Tiraboschi G., Rodrigues da Silva I.C., de Souza W.C. (2020). Increased Sensitivity for Happy Faces in Depressed Patients Following 15 Hz Repetitive Transcranial Magnetic Stimulation (RTMS) over the Left Dorsolateral Prefrontal Cortex. Psychol. Neurosci..

[25] Anderson I.M., Shippen C., Juhasz G., Chase D., Thomas E., Downey D., Toth Z.G., Lloyd-Williams K., Elliott R., Deakin J.F.W. (2011). State-Dependent Alteration in Face Emotion Recognition in Depression. Br. J. Psychiatry.

[26] Rassovsky Y., Dunn W., Wynn J., Wu A.D., Iacoboni M., Hellemann G., Green M.F. (2015). The Effect of Transcranial Direct Current Stimulation on Social Cognition in Schizophrenia: A Preliminary Study. Schizophr. Res..

[27] Keeser D., Meindl T., Bor J., Palm U., Pogarell O., Mulert C., Brunelin J., Moller H.-J., Reiser M., Padberg F. (2011). Prefrontal Transcranial Direct Current Stimulation Changes Connectivity of Resting-State Networks during FMRI. J. Neurosci..

[28] Fonteneau C., Redoute J., Haesebaert F., Le Bars D., Costes N., Suaud-Chagny M.-F., Brunelin J. (2018). Frontal Transcranial Direct Current Stimulation Induces Dopamine Release in the Ventral Striatum in Human. Cereb. Cortex.

[29] Dondé C., Brevet-Aeby C., Poulet E., Mondino M., Brunelin J. (2019). Potential Impact of Bifrontal Transcranial Random Noise Stimulation (TRNS) on the Semantic Stroop Effect and Its Resting-State EEG Correlates. Neurophysiol. Clin..

[30] Kesler-West M.L., Andersen A.H., Smith C.D., Avison M.J., Davis C.E., Kryscio R.J., Blonder L.X. (2001). Neural Substrates of Facial Emotion Processing Using FMRI. Cogn. Brain Res..

[31] Weightman M.J., Air T.M., Baune B.T. (2014). A Review of the Role of Social Cognition in Major Depressive Disorder. Front. Psychiatry.

[32] Solomon D.A., Leon A.C., Endicott J., Mueller T.I., Coryell W., Shea M.T., Keller M.B. (2004). Psychosocial Impairment and Recurrence of Major Depression. Compr. Psychiatry.

[33] Hirschfeld R.M.A., Dunner D.L., Keitner G., Klein D.N., Koran L.M., Kornstein S.G., Markowitz J.C., Miller I., Nemeroff C.B., Ninan P.T. (2002). Does Psychosocial Functioning Improve Independent of Depressive Symptoms? A Comparison of Nefazodone, Psychotherapy, and Their Combination. Biol. Psychiatry.

[34] Moirand R., Imbert L., Haesebaert F., Chesnoy G., Bediou B., Poulet E., Brunelin J. (2022). Ten Sessions of 30 Min TDCS over 5 Days to Achieve Remission in Depression: A Randomized Pilot Study. JCM.

[35] Martin D.M., Teng J.Z., Lo T.Y., Alonzo A., Goh T., Iacoviello B.M., Hoch M.M., Loo C.K. (2018). Clinical Pilot Study of Transcranial Direct Current Stimulation Combined with Cognitive Emotional Training for Medication Resistant Depression. J. Affect. Disord..

[36] Rezaei M., Shariat Bagheri M.M., Ahmadi M. (2021). Clinical and Demographic Predictors of Response to Anodal TDCS Treatment in Major Depression Disorder (MDD). J. Psychiatr. Res..

[37] Mondino M., Szekely D., Bubrovszky M., Bulteau S., Downar J., Poulet E., Brunelin J. (2020). Predicting Treatment Response to 1 Hz RTMS Using Early Self-Rated Clinical Changes in Major Depression. Brain Stimul..

[38] Browning M., Kingslake J., Dourish C.T., Goodwin G.M., Harmer C.J., Dawson G.R. (2019). Predicting Treatment Response to Antidepressant Medication Using Early Changes in Emotional Processing. Eur. Neuropsychopharmacol..

[39] Tranter R., Bell D., Gutting P., Harmer C., Healy D., Anderson I.M. (2009). The Effect of Serotonergic and Noradrenergic Antidepressants on Face Emotion Processing in Depressed Patients. J. Affect. Disord..

[40] Fonteneau C., Mondino M., Arns M., Baeken C., Bikson M., Brunoni A.R., Burke M.J., Neuvonen T., Padberg F., Pascual-Leone A. (2019). Sham TDCS: A Hidden Source of Variability? Reflections for Further Blinded, Controlled Trials. Brain Stimul..

